# Repeatability and reproducibility of the *wzi* high resolution melting-based clustering analysis for *Klebsiella pneumoniae* typing

**DOI:** 10.1186/s13568-020-01164-7

**Published:** 2020-12-14

**Authors:** Ajay Ratan Pasala, Matteo Perini, Aurora Piazza, Simona Panelli, Domenico Di Carlo, Cristian Loretelli, Alessandra Cafiso, Sonia Inglese, Floriana Gona, Daniela Maria Cirillo, Gian Vincenzo Zuccotti, Francesco Comandatore

**Affiliations:** 1 Department of Biomedical and Clinical Sciences “L. Sacco”, Università di Milano, Pediatric Clinical Research Center “Romeo and Enrica Invernizzi”, Milan, Italy; 2 Department of Clinical Surgical Diagnostic and Pediatric Sciences, Microbiology and Clinical Microbiology Unit, University of Pavia, Pavia, Italy; 3 International Center for T1D, Pediatric Clinical Research Center “Romeo Ed Enrica Invernizzi”, Department of Biomedical and Clinical Science L. Sacco, Università di Milano, Milan, Italy; 4 Department of Veterinary Medicine, Università di Milano, Lodi, Italy; 5 Microbiology and Virology Unit, Fondazione IRCCS Policlinico San Matteo, Pavia, Italy; 6 Laboratorio Microbiologia e Virologia-Ospedale San Raffaele Dibit, 2-San Gabriele 1, Milan, Italy; 7 Emerging Bacterial Pathogens Unit, Division of Immunology, Transplantation and Infectious Diseases, IRCCS San Raffaele Scientific Institute, Milan, Italy; 8 Department of Pediatrics, Children’s Hospital Vittore Buzzi, Università di Milano, Milan, Italy

## Abstract

High resolution melting (HRM) is a fast closed-tube method for nucleotide variant scanning applicable for bacterial species identification or molecular typing. Recently a novel HRM-based method for *Klebsiella pneumoniae* typing has been proposed: it consists of an HRM protocol designed on the capsular *wzi* gene and an HRM-based algorithm of strains clustering. In this study, we evaluated the repeatability and reproducibility of this method by performing the HRM typing of a set of *K. pneumoniae* strains, on three different instruments and by two different operators. The results showed that operators do not affect melting temperatures while different instruments can. Despite this, we found that strain clustering analysis, performed using MeltingPlot separately on the data from the three instruments, remains almost perfectly consistent. The HRM method under study resulted highly repeatable and thus reliable for large studies, even when several operators are involved. Furthermore, the HRM clusters obtained from the three different instruments were highly conserved, suggesting that this method could be applied in multicenter studies, even if different instruments are used.

## Introduction

*Klebsiella pneumoniae* is a Gram-negative opportunistic pathogen often present in the gut of healthy individuals but also able to cause severe infections. Furthermore, the bacterium is one of the most important nosocomial pathogens, causing healthcare-acquired infections worldwide with a mortality rate ranging from 20 to 70% (Angus et al. 2001; Mayr et al. 2014). Indeed, *K. pneumoniae* has been described as an “urgent threat to human health” by the United States Centers for Disease Control and Prevention (CDC) and the World Health Organization (WHO) (Munoz-Price et al. 2013). Genomic studies revealed that, despite the high genetic variability of the bacterium (Gaiarsa et al. 2015; Holt et al. 2015), most of the nosocomial outbreaks are caused by only few Multi Drug Resistant (MDR) clones, in particular ST258, ST512, ST307, ST11, ST101 and ST15 (David et al. 2019; Wyres et al. 2019). Thus, a genetic-based nosocomial surveillance can represent an important tool to promptly detect *K. pneumoniae* high risk clones in the hospital setting.

High Resolution Melting (HRM)-based typing is a promising tool for clinical and epidemiological applications (Tamburro and Ripabelli 2017). HRM is a fast closed-tube method to discriminate nucleotide variants on the basis of PCR amplicon melting temperature. The method is particularly reliable for nosocomial surveillance: it can be performed on most real-time PCR instruments; the entire protocol takes ~ 5 h and it is inexpensive (~ 5$ per sample).

Perini and colleagues (Perini et al. 2020a) proposed an HRM-based method for *K. pneumoniae* typing. The method consists of an HRM protocol designed on the hypervariable capsular gene *wzi* and followed by a strains clustering analysis based on the melting temperatures. The method was able to discriminate most of the *K. pneumoniae* Sequence Types (STs) known as “high risk” (Perini et al. 2020b).

Different real-time PCR/HRM instruments can vary in thermal precision and melting temperature acquisition rate (Wittwer 2009; Li et al. 2014). In literature, studies on HRM protocols designed for human samples revealed that the measured melting temperature can vary among the instruments (Wittwer 2009; Li et al. 2014). In this study, we evaluated the repeatability and reproducibility (Schulten et al. 2000; Bustin et al. 2009) of the HRM method described by Perini and colleagues (Perini et al. 2020b) repeating HRM typing on three different instruments by two operators.

## Materials and methods

### Dataset selection

The dataset for the analyses was a subset of the 82-strains collection analyzed by Perini and colleagues (Perini et al. 2020b). Perini and colleagues grouped the 82 strains in a total of seven clusters labelled as: “Cluster ST258 Clade1-like” (including 10 strains), “Cluster ST258 Clade2-like” (27 strains), “Cluster ST11/ST101-like” (22 strains), “Cluster ST307-like” (19 strains), “Cluster ST10/ST147-like” (including two strains), “Cluster ST15-like” (one strain), “Cluster ST149-like” (one strain). To test the repeatability of the protocol we selected a subset of 43 out of the 82 strains, considering the strains from the same cluster as biological replicates. More in detail, we selected 10 strains from the “Cluster ST258 Clade1-like”, 10 from the “Cluster ST258 Clade2-like”, 10 from the “Cluster ST11/ST101-like”, 10 from the “Cluster ST307-like”, one from the “Cluster ST10/ST147-like”, one from the “Cluster ST15-like” and one from the “Cluster ST149-like” cluster.

All the isolates were retrieved from clinical collections and they were isolated from hospital patients (see Additional file 1: Table S1). The 43 *K. pneumoniae* strains belong to eleven different STs, including the highly epidemiologically relevant ST258, ST512, ST307, ST11, ST101 and ST15 (David et al. 2019; Wyres et al. 2019) (see Additional file 1: Table S1 for details).

### Real-time PCR/HRM instruments

HRM analysis (see below) was performed on three different real-time PCR/HRM instruments:

- Bio-Rad CFX96 real-time PCR machine (Bio-Rad Laboratories), from here “CFX96”.
- Eco Real-Time PCR System (Illumina), from here “Eco_RT”.
- QuantStudio 6 Flex Real-Time PCR System (Applied Biosystems), from here “QS_6Flex”.

The three instruments were placed in three different laboratories in two cities, for details see Additional file 2: Table S2.

### DNA extraction

Bacterial strains were freshly streaked on MacConkey agar plate and incubated overnight at 37 °C; then a single colony was inoculated into 5 mL of LB broth (DifcoTM) and incubated overnight at 37 °C with vigorous shaking. For each strain, 1 × 10^9^ cells have been used as starting material for total DNA extraction using the DNeasy blood and tissue kit (Qiagen) following the manufacturer’s instructions.

### High resolution melting analysis

For each strain, the extracted DNA was subjected to six HRM analyses: two operators (AP and MP) independently performed the HRM analyses on the three real-time PCR/HRM instruments listed above. In each of the six HRM analysis, three technical replicates were performed for each strain, amplified with the two primer pairs in the Perini and colleagues (Perini et al. 2020b) HRM protocol (*wzi*-*3* and *wzi*-*4*). Negative controls were added in every HRM analysis for each primer pair.

The HRM reaction mix (10 µl) contained: 5 µl of 2x SsoAdvanced Universal SYBR^®^ Green Supermix (BioRad, Hercules, California), 0.4 µl of each primer (0.4 µM) and 1 µl of template DNA (25–50 ng/µl). The thermal profile was as follows: 98 °C for 2 min, 40 cycles of [95 °C for 7 s, 61 °C for 7 s, and 72 °C for 15 s], 95 °C for 2 min, followed by HRM ramping from 70 to 95 °C. Fluorescence data were acquired at increments of 0.5 °C for CFX96, 0.3 °C QuantStudio 6 Flex, and 0.1 °C for Eco Real-Time PCR System. Each CFX96 and QuantStudio 6 Flex HRM analysis was performed in a single 96-well optical plate, while for the Eco Real-Time PCR System each HRM analysis required three 48-well optical plates.

DNA and reagents aliquots for all the experiments were prepared in advance to reduce the risk of contamination. In each experiment, the two operators independently prepared the HRM mixes in a pre-PCR ‘clean’ room using the same pipettes each day for each individual experiment.

### Statistical analysis

For each strain, the average of the melting temperatures (aTm) obtained from the three technical replicates were computed for *wzi*-*3* and *wzi*-*4* primer sets. A preliminary qualitative comparison of the aTms obtained by the different instruments and operators was performed reporting the median, minimum and maximum temperature differences for *wzi*-*3* and *wzi*-*4* aTms and plotting the aTm distributions by boxplots.

Then, the effects of operators or instruments on *wzi*-*3* and *wzi*-*4* aTms were investigated as independent and as combined factors. The statistical analyses were performed on 1,000,000 bootstrapping strain subsets, randomly selected with replacement. For each subset, aTms were analyzed using R v.3.6.1 (https://www.r-project.org/) as follows:

1. Independent factors:
  Normality distribution of aTm values was tested by the Shapiro–Wilk test.
  Homoskedasticity variances of aTm values between operators and among the three instruments were compared using F test and Bartlett test, respectively.
  If aTms were normally distributed, the operators were compared using *t* test (applying the Welch approximation in case of heteroskedasticity variance), otherwise using Mann–Whitney test.
  If aTms were normally distributed, the instruments were compared using one-way ANOVA (ANalysis Of VAriance, or Welch one-way ANOVA in case of heteroskedasticity variance), otherwise Kruskal–Wallis test.
2. Combined factors:
  The effects of operator and instrument, and their interactions, were evaluated using the non-parametric analysis of variance implemented in the art function of ARTool R 3.6.1 package (Kay 2020).

Then, we evaluated the percentage of subsets for which the effect of operator, instrument or their interaction were significant (p-value < 0.05).

### HRM clustering analysis

For each instrument (CFX96, Eco_RT and QS_6Flex), the obtained melting temperatures were subjected to HRM clustering analysis using the MeltingPlot tool (Perini et al. 2020b) (available at https://skynet.unimi.it/index.php/tools/meltingplot/). More in detail, for each strain the tool computes the replicates average melting temperatures (aTms) for each primer set. Using the igraph R library (http://igraph.org/), the tool builds a graph connecting the strains with aTm distance ≤ 0.5 °C for every primer set, and it clusters the strains on the basis of their betweenness (Perini et al. 2020b). Since the clustering algorithm is based on melting temperature only, the melting curves were not subjected to normalization, smoothing or background adjustment.

## Results

### High resolution melting analysis

Forty-three: *Klebsiella pneumoniae* strains were subjected to the HRM protocol proposed by Perini and colleagues (Perini et al. 2020b) using *wzi*-*3* and *wzi*-*4* primer sets. The analysis was repeated by two operators on three different instruments, for a total of six experiments per strain. The resulting average melting temperatures (aTms) are reported in Additional file 3: Table S3.

### Statistical analysis

**Fig. 1:**
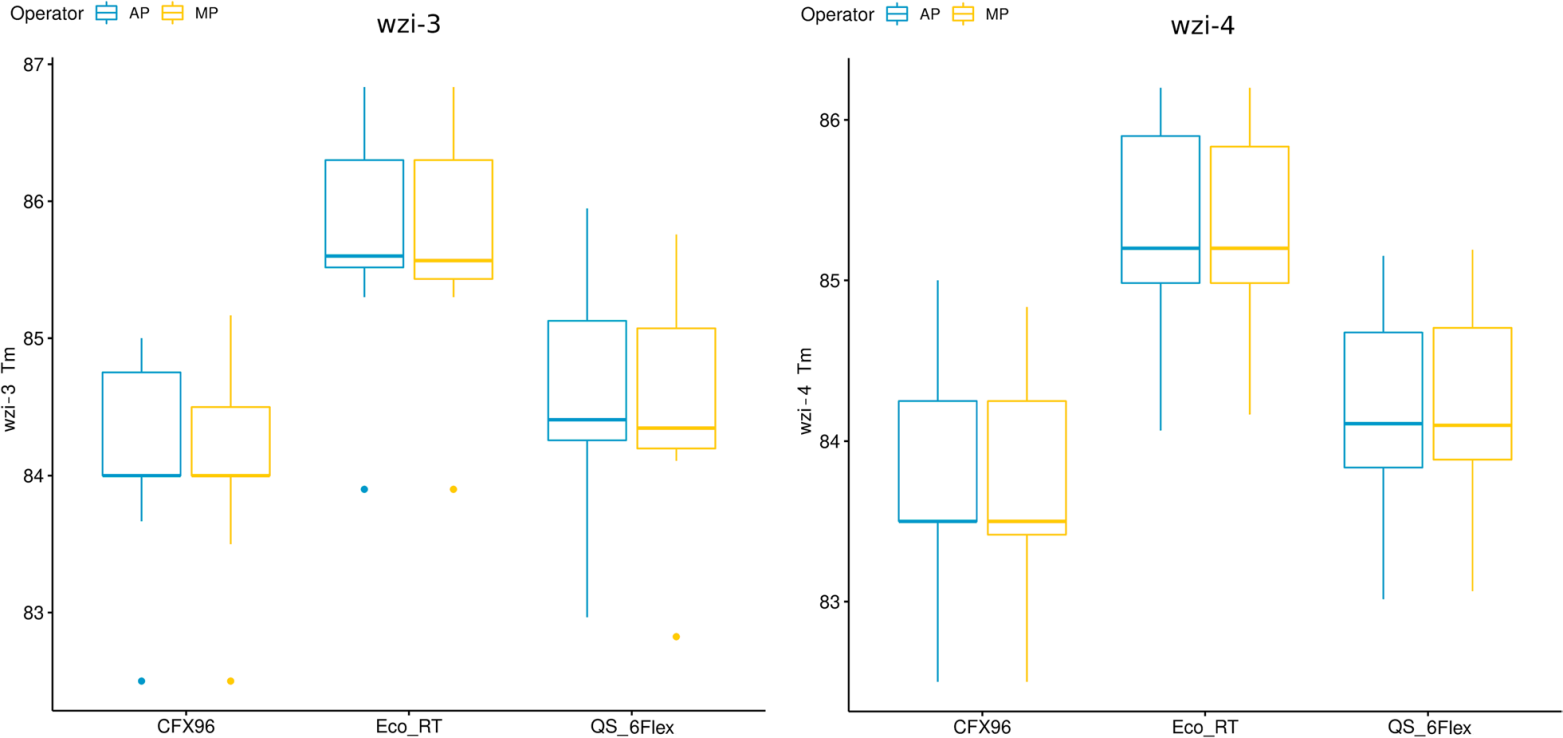
Boxplot of the wzi-3 and wzi-4 average melting temperatures. The distributions of *wzi*-*3* and *wzi*-*4* average melting temperatures (aTm) obtained by each instrument (“CFX96” for Biorad CFX96; “Eco_RT” for Illumina Eco Real-Time; “QS_6Flex” for Applied Biosystem QuantStudio 6 Flex) and operator (AP and MP; colored in blue and yellow, respectively) are shown. Boxes range between the 25th and the 75th quartiles, and bold horizontal lines represent the median values

Boxplots of the aTms from the six HRM experiments are reported in Fig. 1. The median, minimum and maximum aTm differences among operators/instruments are reported in Additional file 4: Table S4, for a total of 15 operator/instrument combinations per primer set.

For either primer sets, the combinations of different operators on the same machine gave a maximum difference below or equal to 0.5 °C, the threshold set by Perini and colleagues (Perini et al. 2020b) for clustering analysis. Conversely, all the combinations among different machines gave maximum differences above 0.5 °C.

The results of the statistical analysis are summarized in Additional file 5: Table S5. For both *wzi*-*3* and *wzi*-*4*, T-test/Wilcox tests on the operators resulted non-significant (p-value ≥ 0.05) for the 100% of the 1,000,000 bootstrap replicates, while the instrument resulted significant (p-value < 0.05) for all of them. For *wzi*-*3*, the non-parametric analysis of the variance found the operator to be significant for 13.37% of the bootstraps replicates, the instrument for 100% of replicates, and the interaction among the two factors (operator/instrument) was never significant among the bootstrap replicates. For *wzi*-*4*, the instrument was found significant for 100% of the bootstrap replicates, while the operator and the operator-instrument interaction were never significant.

### HRM clustering analysis

**Fig. 2:**
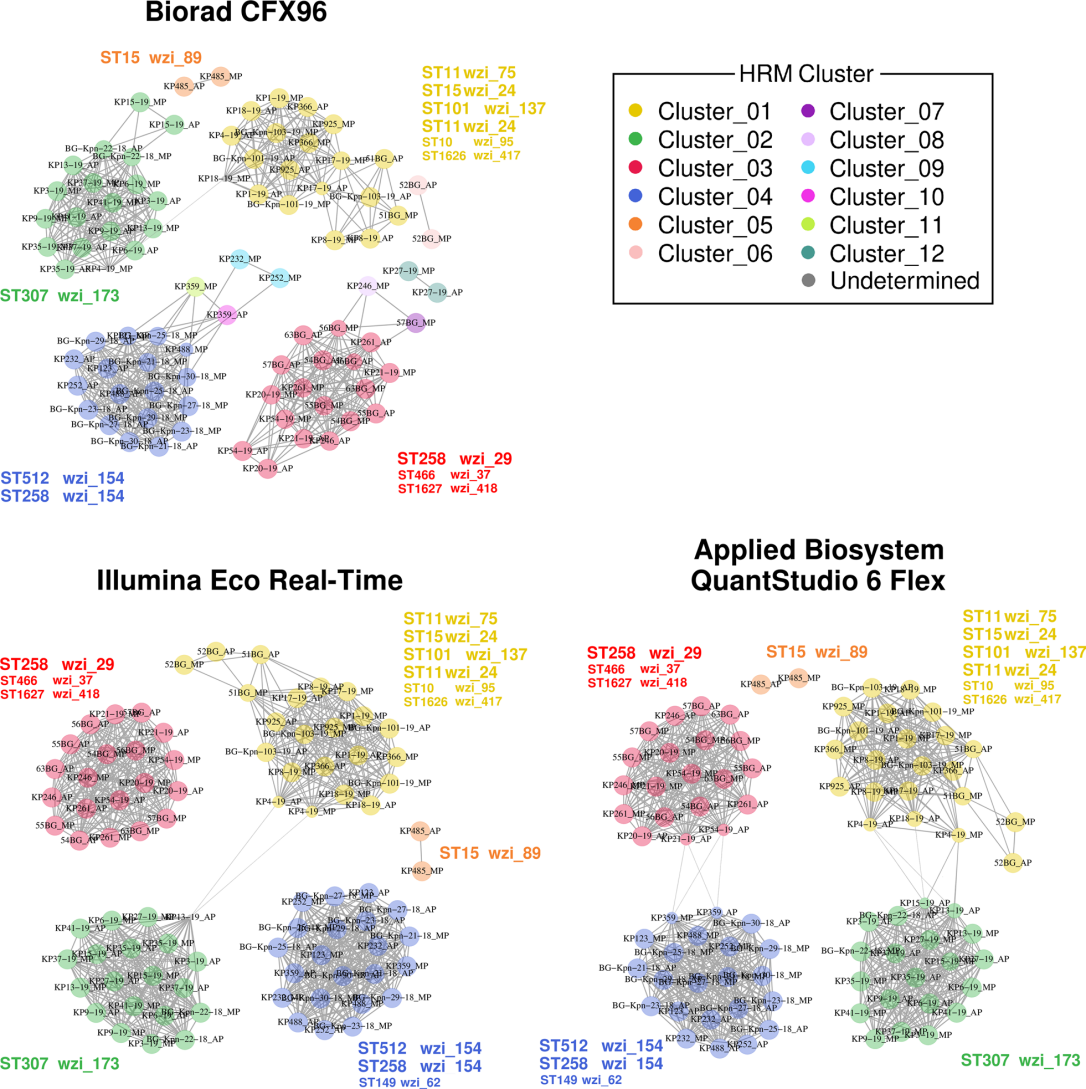
HRM-based strains clustering. The outputs of the MeltingPlot tool (Perini et al. 2020b) on the basis of *wzi*-*3* and *wzi*-*4* melting temperatures obtained by each of the three instruments included in the study (CFX96, ECO_RT and QS_6Flex) are shown. Two strains are connected if the average melting temperatures (aTm) for both *wzi*-*3* and *wzi*-*4* do not differ more than 0.5 °C. The strains clusters were identified by MeltingPlot tool on the basis of graph topology and highlighted by different colors. Genomic information (MLST profile and wzi allele) was added next to each major cluster in the corresponding color. The highly epidemiologically relevant Sequence Types (ST) (David et al. 2019; Wyres et al. 2019) are reported with a bigger font size

**Fig. 3:**
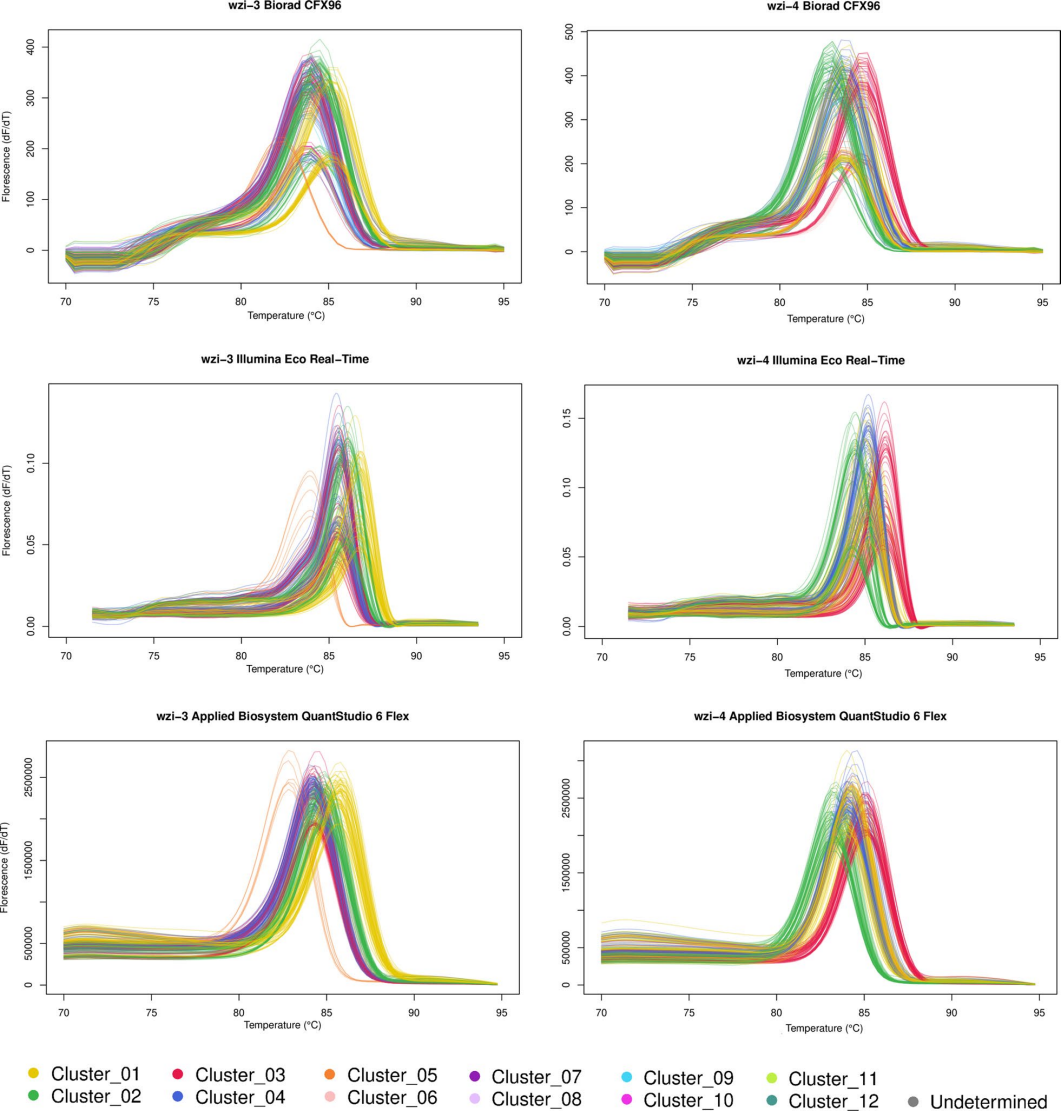
HRM curves. All the raw derivative High Resolution Melting curves obtained for the experiments are reported. The colors represent the clusters found by MeltingPlot tool (Perini et al. 2020b) and correspond to those used in Fig. 2

For each of the three instruments (CFX96, Eco_RT and QS_6Flex), the 43 strains were clustered on the basis of *wzi*-*3* and *wzi*-*4* aTms using the MeltingPlot tool (Perini et al. 2020b). The graph clustering obtained by MeltingPlot is reported in Fig. 2. All the obtained melting curves coloured following the obtained clusters are reported in Fig. 3. The clusters obtained from MeltingPlot analysis on the basis of the melting temperatures obtained from each instrument are reported in Additional file 1: Table S1. For each instrument, the clustering analysis grouped the 43 strains in five main clusters. The strains clusters resulted almost exactly conserved among the instruments. Each of the 43 strains in this work was subjected to HRM analysis twice, one per operator, for a total of 86 instances (each instance is represented by a node in the graph in Fig. 2). The clustering analysis performed using MeltingPlot on the melting temperatures obtained using Illumina Eco Real-Time (ECO_RT) and Applied Biosystem QuantStudio 6 Flex (QS_6Flex), gave the same result for 85 out of 86 instances (98.8%). Indeed, KP13-19 strain for AP operator was classified as Undetermined in the Illumina Eco Real-Time (ECO_RT). On the other hand, the Biorad CFX96 clusters show a total of 10 instances out of 86 (11.6%) that clustered differently from the other two instruments, and two instances (2.3%) classified as Undetermined. More in details, the difference are the following: (i) KP27-19 (for MP operator) and KP27-19 (AP) were assigned to a separate cluster in CFX96 dataset, while to the cluster 04 for the other instruments; (ii) 52BG (MP and AP) were assigned to the cluster 06 in CFX96 dataset, while to cluster 01 for the other instruments. (iii) KP232 (MP), KP252 (MP) were assigned to cluster 09 in CFX96, while to cluster 04 in the other instruments. (iv) KP359 (MP and AP) were assigned to cluster 10 and 11 respectively in CFX96, while they were assigned to cluster 04 in the other instruments. (v) 57BG (MP) and KP246 (MP) were assigned to cluster 07 and 8 respectively in CFX96, while they were assigned to cluster 03 in the other instruments. (vi) KP18-19 (MP) and KP4-19 (MP) were classified as undetermined in the CFX96 while they were classified as cluster 01 in the other instruments.

We compared the clusters obtained by the three instruments to the Perini et al. 2020b clusters, obtained using a CFX96 instrument. As shown in Additional file 6: Table S6, CFX96 experiments from this work clustered 78 instances out of 86 (90.6%) in accordance with Perini et al. 2020b. Among the eight instances differently clustered included, two were classified as Undetermined. The experiments performed using ECO_RT and QS_6Flex grouped the instances in less clusters than Perini et al. 2020b. More in details, both for ECO_RT and QS_6Flex, cluster 01 contains all the instances previously grouped in the Cluster ST10/ST147-like and Cluster ST11/ST101-like clusters, and cluster 04 contains the instances grouped in the Cluster ST149-like and Cluster ST258 Clade2-like. ECO_RT experiments classified one instance as Undetermined. Overall, ECO_RT experiments led to cluster 81 instances out of 86 (94.2%) in accordance with Perini et al. 2020b clusters, while QS_6Flex 84 out of 86 (97.7%).

## Discussion

In the present study we evaluated the repeatability and reproducibility of the *wzi* HRM protocol for *Klebsiella pneumoniae* typing (Perini et al. 2020b). For this validation study, we selected a subset of 43 strains representative of the entire collection of 82 *K. pneumoniae* isolates typed by Perini and colleagues (Perini et al. 2020b). The dataset strains belong to eight different Multi Locus Sequence Typing profiles, including the most epidemiologically relevant clones (David et al. 2019; Wyres et al. 2019), i.e. ST258, ST512, ST307, ST11, ST101 and ST15. In this study we validated the protocol on three real-time PCR/HRM instruments: Biorad CFX96, Illumina Eco Real-Time and Applied Biosystem QuantStudio 6 Flex. The three instruments were placed in three different laboratories in two cities. We also studied the effect of different operators on the results. Two operators (AP and MP) independently performed HRM analysis on the 43 *K. pneumoniae* strains on the three instruments.

Comparing the melting temperatures obtained by the two operators (AP and MP), statistical analyses revealed that the operator does not affect the measured melting temperatures. Furthermore, the operators did not affect the results of clustering analysis (see Fig. 2 and Additional file 1: Table S1): in almost all cases, the same strain analysed independently by the two operators is assigned to the same HRM cluster. This results shows that the HRM protocol proposed by Perini and colleagues (Perini et al. 2020b) is highly repeatable and thus reliable for large scale studies, even if several operators are involved.

Conversely, the instruments resulted to significantly affect the measured melting temperatures (Additional file 5: Table S5). The melting temperatures obtained from the same collection of strains by different instruments can significantly vary (see Fig. 1 and Additional file 4: Table S4). As shown in Additional file 4: Table S4, the differences among the melting temperatures obtained for the same strain by the three instruments often exceed 0.5 °C, the threshold used for the HRM clustering analysis (Perini et al. 2020b). For this reason, melting temperatures obtained using different instruments can not be included in the same HRM clustering analysis. Therefore, a clustering analysis can only be performed using melting temperatures obtained from the same instrument. Nevertheless, the results of the HRM clustering analyses performed on the melting temperatures obtained from three different instruments are almost perfectly conserved (Fig. 2, Fig. 3 and Additional file 1: Table S1). The repeatability of the protocol is also evident comparing the clusters obtained by Perini et al. 2020b with those obtained in this work (Additional file 6: Table S6). The few discrepancies regarded a limited number of strains, while almost all the strains were coherently classified in each of the four independent experiments (Perini et al. 2020b and each of the three instruments used in this work).

The clustering analysing applied by MeltingPlot is based only on the melting temperatures measured by the real time PCR instrument. For this reason the melting curves are not normalized, smoothed or background adjusted as these procedures change the overall shape of the curve but not the melting temperature, i.e. the peak of the derivative melt curve (as in Fig. 3). Despite this approach may cause the loss of information present in the curve, it reduces the influence of experimental noise, thus increasing the repeatability of the obtained results. To compensate this information loss, we developed the HRM protocol on an highly variable gene (*wzi*) and, in particular, on two gene regions rich in HRM detectable SNPs. In this way it is possible to have a wider range of melting temperatures than in HRM typing protocols developed around one or a few specific SNPs.

The main limit of this typing protocol is the low sensitivity of the HRM assay. In particular, using two targets (*wzi*-*3* and *wzi*-*4*) for the HRM assay, the protocol is able to discriminate only strains for which the melting temperatures differ more than 0.5 °C for at least one of the targets. For this reason, strains harbouring different wzi alleles can cluster together (Fig. 2). On the other hand, this limited sensitivity gives robustness to the final results: whenever two strains are clustered separately, they harbour different *wzi* alleles and, therefore, they likely belong to different clones.

In this work we can conclude that the *wzi* HRM protocol is highly repeatable on the same instrument, without significant effect of the operator. Considering the low cost per sample of this protocol (~ 5$ per sample) and short time required to accomplish the analysis (~ 5 h), it is a typing method suitable for the real time monitoring of the epidemiological scenario in a hospital setting. A day by day monitoring of the *K. pneumoniae* clones circulating in a hospital can allow the prompt detection of the emergence of a nosocomial outbreak and to follow the spreading of the outbreak clone among the patients/wards.

## Supplementary information

**Additional file 1: Table S1.** Strains genomic information and Results of clustering analysis. For each strain included in the study the cluster assigned by MeltingPlot tool on the basis of *wzi*-*3* and *wzi*-*4* melting temperatures obtained by each instrument and operator (“CFX96” for Biorad CFX96; “Eco_RT” for Illumina Eco Real-Time; “QS_6Flex“for Applied Biosystem QuantStudio 6 Flex) are reported. The relative hospital of isolation (Hospital), Multi Locus Sequence Typing (MLST) profiles, the *wz*i alleles (*wzi*) and the clusters described in Perini et al. 2020b (Perini et al. 2020b) (Perini et al. 2020b Clusters)are also reported.

**Additional file 2: Table S2.** Instruments information. For each instrument used in this work, the model, the short name (used in manuscript, tables and figure), the sensitivity and the location are reported.

**Additional file 3: Table S3.** Strains melting temperatures. The instrument used for the HRM analysis, the operator who performed the HRM analysis, the primer set used, the melting temperature replicates (T1, T2 and T3) and their average melting temperature (aTm) are reported for each strain.

**Additional file 4: Table S4**. Melting temperature differences among instruments and operators. The median of the average melting temperatures differences for each strain among instruments and operators are reported. In brackets, minimum and maximum difference values are reported. The comparisons between the two operators on the same instrument are reported in bold.

**Additional file 5: Table S5.** Results of statistical analyses. The results of statistical analyses are reported.

**Additional file 6: Table S6.** Cross table of the clusters found in Perini et al. 2020b and the clusters of each instrument used in this work is reported. The discrepancies from the clusters found in Perini et al. 2020b are reported in bold.

## Data Availability

The isolates analysed in this work is a subset of an isolate collection analysed also in a published work (Perini et al. 2020b): Short Read Archive ERP119329; BioProject PRJEB36171. MeltingPlot, the tool used for the clustering analysis, is available online at https://skynet.unimi.it/index.php/tools/meltingplot/.
